# The Single Nucleotide Polymorphism Gly482Ser in the PGC-1α Gene Impairs Exercise-Induced Slow-Twitch Muscle Fibre Transformation in Humans

**DOI:** 10.1371/journal.pone.0123881

**Published:** 2015-04-17

**Authors:** Peter Steinbacher, René G. Feichtinger, Lyudmyla Kedenko, Igor Kedenko, Sandra Reinhardt, Anna-Lena Schönauer, Isabella Leitner, Alexandra M. Sänger, Walter Stoiber, Barbara Kofler, Holger Förster, Bernhard Paulweber, Susanne Ring-Dimitriou

**Affiliations:** 1 Department of Cell Biology, Paris Lodron-University of Salzburg, Salzburg, Austria; 2 Research Program for Receptor Biochemistry and Tumor Metabolism, Department of Pedicatrics, Paracelsus Medical University of Salzburg, Salzburg, Austria; 3 First Department of Internal Medicine, Paracelsus Medical University of Salzburg, Salzburg, Austria; 4 Medical Office in Pediatrics and Sports Medicine, Salzburg, Austria; 5 Department of Sport Science and Kinesiology, Paris Lodron-University of Salzburg, Hallein, Austria; University of Louisville School of Medicine, UNITED STATES

## Abstract

PGC-1α (peroxisome proliferator-activated receptor γ co-activator 1α) is an important regulator of mitochondrial biogenesis and a master regulator of enzymes involved in oxidative phosphorylation. Recent evidence demonstrated that the Gly482Ser single nucleotide polymorphism (SNP) in the *PGC-1α* gene affects insulin sensitivity, blood lipid metabolism and binding to myocyte enhancer factor 2 (MEF2). Individuals carrying this SNP were shown to have a reduced cardiorespiratory fitness and a higher risk to develop type 2 diabetes. Here, we investigated the responses of untrained men with the Gly482Ser SNP to a 10 week programme of endurance training (cycling, 3 x 60 min/week, heart rate at 70-90% VO_2peak_). Quantitative data from analysis of biopsies from *vastus lateralis* muscle revealed that the SNP group, in contrast to the control group, lacked a training-induced increase in content of slow contracting oxidative fibres. Capillary supply, mitochondrial density, mitochondrial enzyme activities and intramyocellular lipid content increased similarly in both groups. These results indicate that the impaired binding of MEF2 to PGC-1α in humans with this SNP impedes exercise-induced fast-to-slow muscle fibre transformation.

## Introduction

Skeletal muscle is a tissue with excellent plasticity in response to external stimuli such as exercise and training. The repetitive muscle contractions conducted during endurance training lead to a variety of phenotypic and physiological responses. These responses include activation of mitochondrial biogenesis, fibre type transformation and angiogenesis. Together, they increase the muscle’s capacity of aerobic metabolism and its resistance to fatigue. At the whole-body level, these adaptive changes are the basis for the improvement of physical performance and other health benefits [1]. Regular endurance training is therefore a common strategy to reduce high blood pressure and to prevent cardiovascular and metabolic diseases such as type 2 diabetes mellitus (T2D) [2–9].

Mammalian skeletal muscle consists of different muscle fibre types, each with different contraction speed, type of energy metabolism, and amounts of cell organelles [10]. These fibre types are also distinguished by their myosin heavy chain (MHC) isoform composition. Oxidative slow-twitch type I fibres (henceforth briefly called ‘slow fibres’) contain MHC-Iβ. They use oxidative phosphorylation (OXPHOS) to generate ATP and are thus highly fatigue resistant and preferentially activated during endurance exercise. Slow fibres comprise high amounts of mitochondria, myoglobin and lipid droplets, and are well supplied by capillaries. In addition, there are three types of fast-twitch fibres (types IIA, IID/X, IIB, with the corresponding MHC isoforms IIa, IId/x, IIb) which are all used for rapid high-force generation. Oxidative-glycolytic fast-twitch type IIA fibres have intermediate amounts of mitochondria, lipid droplets and capillaries, and are intermediately resistant to fatigue (as compared to type I and types IIB and IID/X). Glycolytic fast-twitch type IID/X fibres are poor in mitochondria, lipids and capillaries and more susceptible to fatique than type IIA. Glycolytic fast-twitch type IIB fibres have the lowest amounts of mitochondria, lipid droplets and capillaries, but generate the highest contraction velocities [10].

Important responses to endurance training occur at the intracellular level. They include increases in size and number of mitochondria as well as such in the activities of oxidative enzymes [11,12,1]. In support of the increased oxidation of fatty acids, the content of intramyocellular lipid is also elevated [13].

In rodents and humans, it has been demonstrated that peroxisome proliferator-activated receptor gamma coactivator-1 alpha (PGC-1α) is implicated in the regulation of the exercise-induced changes of muscle fibres towards a slow phenotype, as well as in the protection of muscle atrophy [12,14,15]. Activation of PGC-1α has been shown to regulate lipid and carbohydrate metabolism, and to improve the oxidative capacity of the muscle fibres by increasing the amount and activity of mitochondria through upregulation of nuclear respiratory factors (NRF-1, 2) and mitochondrial transcription factor A (TFAM) [16,17]. PGC-1α also regulates genes involved in the determination of muscle fibre type. Overexpression of PGC-1α increases the proportion of oxidative type I fibres [18] while PGC-1α knock-out (KO) mice exhibit a shift from oxidative type I and IIA toward type IID/X and IIB fibres [19]. This regulatory diversity of PGC-1α is enabled by its broad binding capacity to transcription factors in various signalling pathways.

Several studies have shown that PGC-1α is upregulated after endurance training [20–24], and also after acute 3h bouts of cycling on consecutive days [25]. The cycling bouts additionally evoked acute increases in cytochrome c oxidase (COX), citrate synthase (CS), and sirtuin 1 (SIRT1), a nicotinamide adenine dinucleotide (NAD)+-sensing protein that deacetylates PGC-1α [25]. In this context, Wright et al. [26] suggested that an exercise-induced activation of p38 mitogen-activated protein kinase (MAPK) phosphorylates the PGC-1α protein before it is transferred from the cytosol into the myonucleus. Via coactivation of transcription factors and nuclear receptors, PGC-1α then mediates the initial phase of the exercise-induced increase in mitochondria. The subsequent upregulation of PGC-1α expression enhances and/or maintains mitochondrial biogenesis, eventually leading to an increased mitochondrial content of the muscle fibres.

PGC-1α also plays an important role in the pathogenesis of insulin resistance and T2D [27]. Furthermore, it has been demonstrated that subjects with a family history of T2D or manifest T2D are characterised by a significantly lower expression of PGC-1α in muscle cells compared to overweight healthy subjects with no family history of T2D [28]. Similarly reduced mRNA levels of skeletal muscle PGC-1α in sedentary T2D subjects have been found by Timmons et al. [29], indicating diminished substrate oxidation.

In human populations, the PGC-1α gene exists in several variants which differ in a single nucleotide from the major allele. Among these single nucleotide polymorphisms (SNPs), it is particularly the Gly482Ser SNP which has been intensively studied. This SNP is known to be less frequent in endurance athletes than in untrained persons [30,31]. Subjects that carry this gene variant are more likely to have increased levels of low-density cholesterol [32] and higher insulin resistance [33]. In response to training, such persons show a lower increase in individual anaerobic threshold and insulin sensitivity than subjects without this SNP [34]. As a result, carriers of the Gly482Ser SNP have a reduced cardiorespiratory fitness and a higher risk for metabolic syndrome and T2D [35,33,30,36,37]. In a previous study, we were able to show that the trainability of middle-aged male carriers of this SNP is reduced, particularly when measured at submaximal exercise performance markers such as the respiratory compensation point (RCP) [38]. However, the background at the molecular level is largely unknown. The work of Choi et al. [39] provides indication that the Gly482Ser gene variant impairs the transcription of TFAM, which plays a role in mtDNA replication.

In the present study, we investigated the myocellular responses in the vastus lateralis muscle of untrained male carriers of this SNP and of a control group to a 10 weeks programme of endurance training (cycling, 3 x 60 min/week, heart rate at 70–90% VO_2peak_). In particular, it was tested how the training influences muscle fibre type proportions, capillary density, content of mitochondria and intramyocellular lipid (IMCL), and the activities of respiratory chain enzymes (CS, OXPHOS complexes (COX) I-V).

## Materials and Methods

### Subjects and experimental design

The participants of the study were recruited from the 1770 persons of the SAPHIR (Salzburg Atherosclerosis Prevention Programme in Subjects at High Individual Risk) study population, all of which were genotyped for the SNP Gly482Ser (rs8192678) in the gene *PPARGC1A* [40]. 28 males (age 59±7 years, range 50–69) from this population volunteered to participate in the present investigation (Table 1). The inclusion criteria set were: males, aged 50–65 years, no history of participation in organized sports, level of regular physical activity ≤ 1 h/week, low cardiorespiratory fitness, no chronic illness (including manifest T2D), no surgery within the last six months. All persons have given written informed consent before entering the study. The study was conducted with permission of the Ethics Committee of the Federal State of Salzburg (E1243, 2010-10-04). Anthropometric parameters (age, height, weight) were determined at the beginning and at the end of the exercise intervention. Regional body fat was determined by measuring the waist circumference 0.5 cm below the umbilicus with a standardized spring-loaded elastic tape (Roche, Germany) in standing upright position with feet 20–30 cm apart [41].

**Table 1:** Baseline characteristics of whole sample and genotypes.

Groups	Total	GT1	GT2	
SNP-*PPARGC1A*	G/A, G/A, A/A	G/G (Gly/Gly)	X/A (X/Ser)	*p*
Sample size, *n*	28	13	15	
Age (yrs)	58.7 ± 1.2	59.3 ± 2.1	58.1 ± 1.5	ns
BM (kg)	88.0 ± 2.2	89.9 ± 4.2	86.3 ± 2.0	ns
BMI (kg m^-2^)	27.8 ± 0.8	28.2 ± 1.4	27.4 ± 3.1	ns
Waist (cm)	101.0 ± 1.9	101.7 ± 3.5	100.4 ± 2.0	ns
SF proportion (%)	55.5 ± 2.1	50.5 ± 2.5	59.9 ± 2.8	0.02
HF proportion (%)	1.5 ± 0.2	1.5 ± 0.3	1.4 ± 0.3	ns
Cap/SF	6.50 ± 0.13	6.37 ± 0.15	6.61 ± 0.20	ns
Mito-SF (%)	7.47 ± 0.25	6.97 ± 0.36	7.90 ± 0.33	ns
Mito-FF (%)	3.53 ± 0.16	3.35 ± 0.19	3.67 ± 0.25	ns
Lip-SF (%)	1.19 ± 0.13	1.32 ± 0.21	1.07 ± 0.16	ns
Lip-FF (%)	0.39 ± 0.05	0.41 ± 0.10	0.37 ± 0.05	ns
mtDNA copies/cell	4212 ± 355	4095 ± 401	4313 ± 578	ns
CS [mU/mg prot.]	251 ± 17	259 ± 22	244 ± 26	ns
C I [mU/mg prot.]	39 ± 2	41 ± 3	36 ± 4	ns
C II [mU/mg prot.]	63 ± 5	63 ± 7	62 ± 8	ns
COX [mU/mg prot.]	280 ± 23	312 ± 35	252 ± 30	ns
C V [mU/mg prot.]	84 ± 10	89 ± 15	80 ± 15	ns
Complex I/CS	0.16 ± 0.01	0.17 ± 0.01	0.15 ± 0.01	ns
Complex II/CS	0.26 ± 0.02	0.25 ± 0.02	0.26 ± 0.03	ns
COX/CS	1.17 ± 0.09	1.27 ± 0.13	1.08 ± 0.12	ns
Complex V/CS	0.32 ± 0.03	0.31 ± 0.04	0.32 ± 0.05	ns

Values are means ±S.E. after one-way ANOVA; GT1 = major allele type in *PPARGC1A* (G/G, wild type), GT2 = homozygous and heterozygous for minor allele frequency in *PPARGC1A* (A/A, G/A); BM = body mass, BMI = body mass index; VO_2-RCP_ = oxygen uptake at respiratory compensation point (RCP, second ventilatory threshold, aerobic capacity), VO_2-peak_ = peak oxygen uptake at cessation (aerobic power), P_RCP_ = mechanical power at RCP in Watt, P_max_ = maximum mechanical power in Watt; SF = slow fibre (MHC slow+/MHC fast-); HF = slow-fast hybrid fibre (MHC slow+/MHC fast+); cap/SF = capillaries per slow fibre; Mito-SF = mitochondria in slow fibre; Mito-FF = mitochondria in fast fibre; Lip-SF = lipid droplets in slow fibre; Lip-FF = lipid droplets in fast fibre; mtDNA = mitochondrial DNA; CS = citrate synthase; C I—V = complex I—V; COX = cytochrome c oxidase; *p* = significance level between the genotype (GT) groups; *ns* = not significant.

According to gene analysis [38], subjects were assigned to two groups: (i) genotype 1 (GT1)—control group carrying the major frequency allele type (*PPARGC1A*, rs8192678 G/G), 13 subjects; (ii) genotype 2 (GT2)—test group carrying the Gly482Ser SNP (minor allele type *PPARGC1A*, rs8192678 x/A), 15 subjects.

All subjects performed a fully supervised 10 weeks cycling training, 3x 60 min/wk at a heart rate equalling 70% to 90% of peak oxygen uptake (VO_2peak_). Trainability was determined as the change in work rate at the respiratory compensation point (P@RCP) based on gas exchange analyses (ZAN680, nSpire Health) during incremental cycling (ergoselect 100, Ergoline, GER), and as the change in cardiorespiratory fitness (VO_2peak_). A ramp test protocol on an electronically-braked bicycle ergometer (ergoselect 100, Ergoline, GER) with 10 W increments per minute (60 to 70 rpm) was applied to determine RCP and VO_2peak_ [42–44]. In each bout of training, each participant started with a warm-up cycling at 50 W over 4 minutes. Volitional fatigue was reached as defined by the following criteria: 90% of age-adjusted maximum heart rate, VO_2peak_ change ≤ 2 ml∙min^-1^∙kg^-1^ with increasing load, and respiratory exchange ratio of ≥1.1. According to the utilized protocol, VO_2peak_ was assessed in the last stage as the mean value of five consecutive breaths with the third breath including the maximal VO_2_-value (for further details see [38]). Parallel to exercise testing, heart rate (HR) was registered with a beat-to-beat monitoring system (T4, Suunto, FIN) to determine exercise training intensity set at a heart rate equaling the pre-training work rate below and at the RCP. The RCP was determined by the disproportional increase of minute ventilation (V_E_) versus carbon dioxide production (VCO_2_) according to Wassermann et al. [45] (details described in [38]).

### Muscle biopsies and tissue preparation

Resting needle biopsies (needle diameter 5 mm) were taken from the *M*. *vastus lateralis* of the non-dominant leg using the percutaneous needle biopsy technique [46]. Functional leg dominance was determined by single-leg voluntary isometric contraction against a force-platform. Biopsies were performed under local anaesthesia seven days before first training (pre-sample), and seven days after last training (post-sample). Each tissue sample was dissected such that specimens of sufficient size and quality were available for the following procedures: (i) One specimen was longitudinally oriented on a small strip of aluminium foil, coated with Tissue-Tek O.C.T. compound cryostat embedding medium (Sakura), cryofixed by plunging in isopentane (2-methylbutane) cooled to near its freezing point, and stored in liquid nitrogen until used for immunocytochemistry. (ii) A second specimen was immersion-fixed in cacodylate-buffered 2.5% glutaraldehyde (overnight, 4°C), postfixed in 1% OsO_4_ (3 h, at room temperature), and embedded in glycid ether-100 epoxy resin for semithin section analysis and electron microscopy according to standard procedures [47]. (iii) Surplus tissue was frozen in liquid nitrogen in small vials and employed for measurement of mitochondrial enzyme activities.

### Immunocytochemistry

Tissue-Tek-coated specimens were cut into serial transverse sections (10 μm) on a Leitz 1720 cryostat. Sections were collected on poly-L-lysine coated slides, dried for 1 h at room temperature and stored at—30°C until further processed. Indirect immunostaining served to identify fibre type-specific MHC isoforms and endothelial cells. Monoclonal anti-rabbit MHC fast IgG1 (1:400, Sigma M 4276), anti-human MHC slow IgG1 (1:4000, Sigma M 8421) and anti-human CD31 IgG1 (1:20, Dako M0823) served as primary antibodies. HRP-conjugated anti-mouse serum (1:100, Dako) was applied as secondary antibody. All antibodies were diluted with PBT-B-N (PBT containing 2% BSA and 5% goat serum). For labelling, sections were fixed in acetone (–20°C), washed in PBT (3x3 min), and blocked with PBT-B-N (5 min); this was followed by primary antibody incubation (1–3 h), 3x3 min washing in PBT, blocking in PBT-B-N (5 min), and secondary antibody incubation (30–60 min). After washing in PBT (3x3 min), antibody binding was visualized with DAB (diaminobenzidine-dihydrochloride). Sections were mounted in Gel/Mount (Biomeda). Photographs of the results were taken on a Reichert Polyvar microscope. A minimum of 300 fibres per sample was evaluated for analysis of fibre type distribution on anti-MHC stained sections. Relative numbers of slow/type I (MHC slow+/MHC fast-) and fast/type II (MHC slow-/MHC fast+) fibres were evaluated. In addition, slow-fast hybrid fibres (MHC slow+/MHC fast+) were also analysed. Sections immediately neighboured to those used for fibre type evaluation were stained for CD31 and served to determine the numbers of capillaries per slow fibre. Numbers of CD31-positive capillaries adjoining to the surface contours of 30 randomly selected slow fibres per individual were counted. To prevent bias by less capillarised adjacent fast fibres, only such slow fibres were examined, which were predominantly (> 75% of their contour) bordered by other slow fibres.

### Electron microscopy and stereological analysis

Transverse and longitudinal semithin (1 μm) and ultrathin (70–90 nm) sections were cut from the epoxy resin embedded specimens on a Leica Ultracut microtome. Semithin sections were stained with azure II-methylene blue and digitally photographed through a Reichert Polyvar microscope. Ultrathin sections were mounted on 75-mesh copper grids, contrasted with uranyl acetate and lead citrate (Leica EM-Stain) and viewed in a Zeiss EM 910 transmission electron microscope (TEM) at 80 kV. A Tröndle Sharp:Eye digital camera system served for documentation and photosampling.

TEM-based photosampling for stereological analysis of intracellular components was undertaken for slow/type I and fast/type II fibres. Slow/type I fibres were identified at overview magnifications by their higher content of lipid droplets and subsarcolemmal mitochondria. Previous work confirms that this allows a sufficiently reliable distinction of type I fibres from all type II fibres for purposes of stereological analysis of intracellular components [47–50]. Fibres with extremely low content of mitochondria and lipid droplets were regarded as belonging to the fast type IIX/D (for identification criteria see [10]). Such fibres were scarce and excluded from further analysis, so that the investigated fast/type II fibres were, in all probability, type IIA fibres. A minimum of six randomly chosen muscle fibres of each type per specimen were analysed. Ten equidistant non-overlapping images per fibre (5 of central areas, 5 of subsarcolemmal areas) were taken at a magnification of x3150. Relative volumes (volume densities) of mitochondria and intramyocellular lipid (IMCL) were determined by point counting using a coherent test system [51]. Volume densities (V_V_) expressed as percentages were derived from: V_V_ = V_α_/V_R_ = P_α_/P_R_ x 100, whereby V_α_ is the volume of the measured cell component, P_α_ is the number of test points falling on this component, V_R_ is the volume of the reference system (section, s), and P_R_ is the number of test points falling on the reference system (in this case the analysed muscle fibre).

### Spectrophotometric measurement of OXPHOS enzyme- and citrate synthase activity

Muscle samples (20–100 mg) were homogenized with a tissue disintegrator (Ultraturrax, IKA, Staufen, Germany) in extraction buffer (20 mM Tris-HCl, pH 7.6, 250 mM sucrose, 40 mM KCl, 2 mM EGTA) and finally homogenized with a motor-driven Teflon-glass homogenizer (Potter S, Braun, Melsungen, Germany). The homogenate was centrifuged at 600*g* for 10 min at 4°C. The postnuclear supernatant (600*g* homogenate) containing the mitochondrial fraction was used for measurement of enzyme activities.

Spectrophotometric measurement of the OXPHOS enzyme- and citrate synthase activity was performed as previously described [52,53]. Citrate synthase was determined according to Srere [54], with modifications. Briefly, the reaction mixture contained 50 mM Tris- HCl pH 8.1, 0.1% bovine serum albumin (BSA), 0.1% TritonX-100, 0.2 mM 5,5’-dithio-bis(2-nitrobenzoic acid), 0.15 mM acetyl-CoA and the 600g homogenate. After initially recording thiolase activity for 2 min the citrate synthase reaction was started by addition of 0.5 mM oxaloacetate and was followed at 412 nm for 8 min.

Enzyme activities of the OXPHOS complexes were determined as previously described [55,56]. Briefly, rotenone-sensitive complex I activity was measured spectrophotometrically as NADH/decylubiquinone oxidoreductase at 340 nm. The enzyme activities of citrate synthase and complex IV (ferrocytochrome c/oxygen oxidoreductase), and the oligomycin-sensitive ATPase activity of the F_1_F_0_ ATP synthase were determined by using buffer conditions as previously described by Rustin et al. [57]. The whole reaction mixture for the ATPase activity measurement was treated for 10 seconds with an ultra-sonifier (Bio cell disruptor 250, Branson, Vienna, Austria). Complex II activity was measured according to Rustin et al. [57] with the following modifications. The reaction mixture contained 50 mM potassium phosphate pH 7.8, 2 mM EDTA, 0.1% BSA, 3 μM rotenone, 80 μM 2,6-dichlorophenol, 50 μM decylubiquinone, 1 μM antimycin A, 0.2 mM ATP, 0.3 mM KCN and the 600g homogenate. The mixture was preincubated for 10 min at 37°C, started by addition of 10 mM succinate, and followed for 6 min at 600 nm.

The reaction mixture for the measurement of the complex III activity contained 50 mM potassium phosphate buffer pH 7.8, 2 mM EDTA pH 8.6, 0.3 mM KCN, 100 μM cytochrome c, 200 μM reduced decyl-ubiquinol. The reaction was started by addition of the 600g homogenate. After 3–4 min the reaction was inhibited by addition of 1 μM antimycin A. Antimycin A- insensitive activity was substracted from total activity to calculate complex III activity. All spectrophotometric measurements (Uvicon 922, Kontron, Milan, Italy) were performed at 37°C.

### Statistics

Data are given as mean ± SE. Shapiro-Wilk tests were conducted to examine data sets for normal distribution. Inter-group differences at baseline were tested for statistical significance using ANOVA. Differences between measurements before and after training within groups were tested with a paired t-test. Interaction of genotype and exercise training was tested with ANOVA for repeated measurements (GT-groups as between factor, time as within factor) with age, body mass and waist as covariates. Differences were considered significant at p ≤ 0.05. Statistical analyses were carried out using Statistical Package for Social Sciences (SPSS 20.0, Chicago, IL, USA).

## Results

All participants responded to the training programme by a significant decrease in body weight by 1.3 ± 0.3 kg (i.e. -1.5%), and a decrease of the BMI of 0.4 kg m^-2^ (-0.4%, Table 2).

**Table 2:** Effect of genotype and 10-weeks supervised stationary cycling on anthropometric variables, aerobic capacity and power, myocellular variables and mitochondria enzyme activity.

Groups	Total	GT1	GT2	
SNP-PPARGC1A		G/G (Gly/Gly)	A/A (X/Ser)	*p*
*n*	28	13	15	
BM (kg)	-1.3 ± 0.3***	-1.2 ± 0.5*	-1.3 ± 0.5*	ns
BMI (kg m^-2^)	-0.4 ± 0.1***	-0.4 ± 0.1*	-0.4 ± 0.2*	ns
Waist (cm)	-0.7 ± 0.5	-1.3 ± 0.8	-0.2 ± 0.4	ns
SF proportion (%)	3.3 ± 2.1	8.9 ± 2.6**	-1.5 ± 2.6	0.01
HF proportion (%)	-0.4 ± 0.3	-0.6 ± 0.5	-0.2 ± 0.3	ns
Cap/SF	1.18 ± 0.07***	1.19 ± 0.10***	1.17 ± 0.10***	ns
Mito-SF (%)	2.71 ± 0.42***	2.99 ± 0.50***	2.46 ± 0.66**	ns
Mito-FF (%)	1.73 ± 0.32***	2.35 ± 0.47***	1.24 ± 0.41**	ns
Lip-SF (%)	0.89 ± 0.19***	1.14 ± 0.26***	0.68 ± 0.28*	ns
Lip-FF (%)	0.10 ± 0.09	0.19 ± 0.15	0.02 ± 0.11	ns
mtDNA copies/cell	122 ± 377	90 ± 323	149 ± 658	ns
CS [mU/mg prot.]	108 ± 28***	152 ± 48**	71 ± 31*	ns
C I [mU/mg prot.]	-0.1 ± 5	1.5 ± 8	-1.6 ± 7	ns
C II [mU/mg prot.]	21 ± 7**	26 ± 9**	16 ± 10	ns
COX [mU/mg prot.]	294 ± 54***	356 ± 89**	240 ± 64**	ns
C V [mU/mg prot.]	43 ± 25	50 ± 34	37 ± 37	ns
Complex I/CS	-0.05 ± 0.02**	-0.06 ± 0.02**	-0.05 ± 0.02	ns
Complex II/CS	0.00 ± 0.02	-0.00 ± 0.03	-0.00 ± 0.03	ns
COX/CS	0.44 ± 0.12***	0.36 ± 0.14*	0.51 ± 0.19*	ns
Complex V/CS	0.00 ± 0.05	0.00 ± 0.06	-0.01 ± 0.08	ns

Values are paired differences ± SE from paired sample *t*-Test after testing with general linear model for repeated measures; *p* = significant interaction between time and genotype; *p≤.05, **p≤.01 and ***p ≤.001 indicate significant differences of pre vs. post measures within group; ns, not significant; for other abbreviations see Table 1.

### Expression of fibre type specific proteins

**Fig 1:**
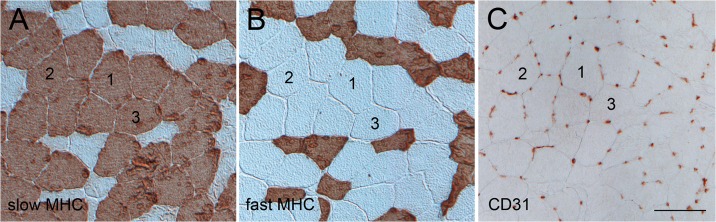
Fibre type composition and capillarisation. Example of serial cross-sectional images of *vastus lateralis* muscle biopsies immunostained for (A) slow myosin heavy chain (MHC), (B) fast MHC, and (C) the endothelial cell marker CD31. Numbers mark identical slow muscle fibres in the section series. Scale bar: 50 μm.

**Fig 2:**
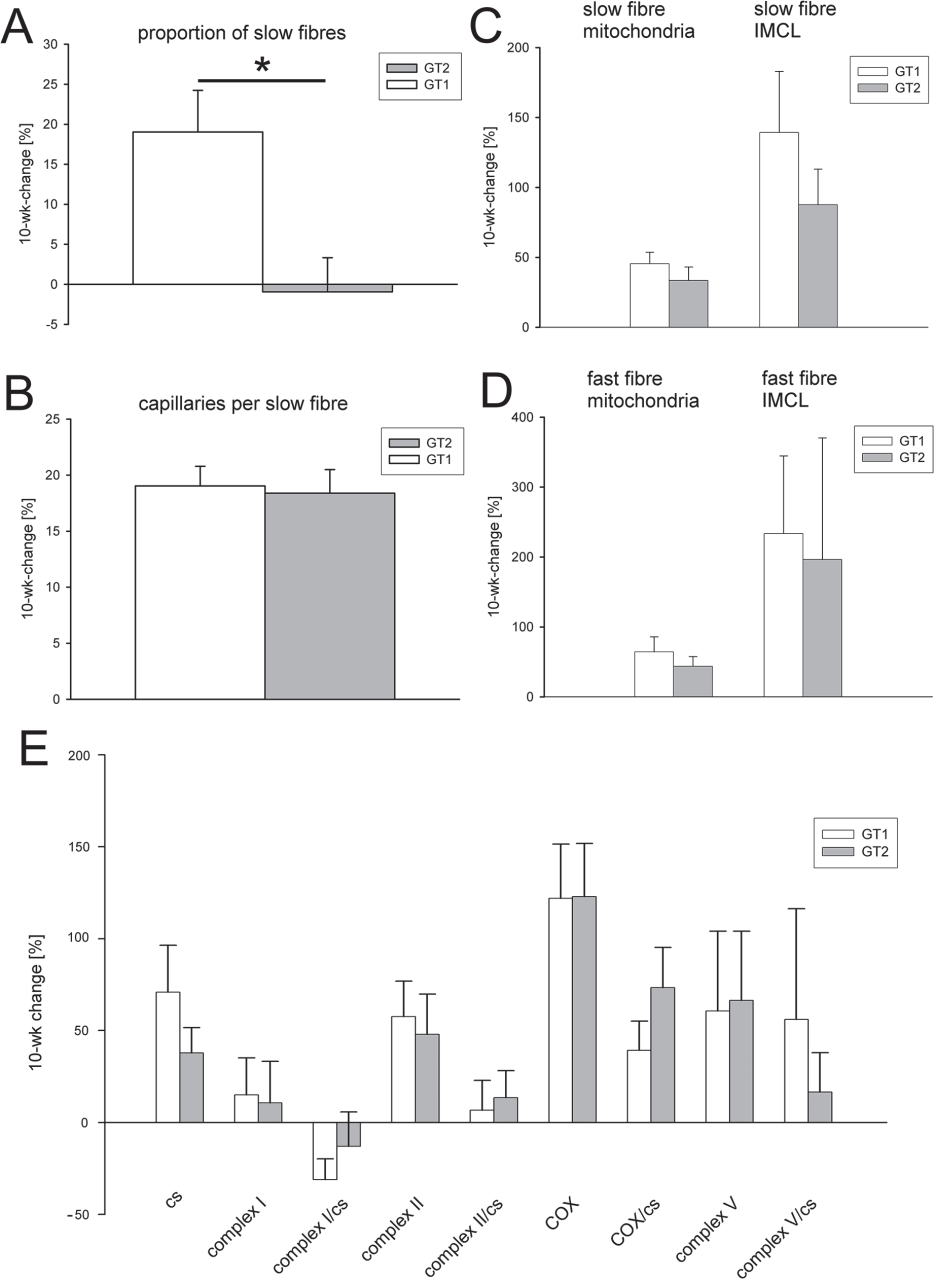
Relative changes [%] of muscle characteristics in response to the 10 weeks endurance training programme. (A) Control individuals (GT1) show a significant increase of the slow fibre proportion after training while carriers of SNP Gly482Ser (GT2) are unaffected. (B) There is no difference between GT1 and GT2 in the increase of capillaries. (C) Controls (GT1) and SNP carriers (GT2) exhibit no significant differences in the training-induced increases in volume density of slow fibre mitochondria and intramyocellular lipid (IMCL). (D) The increases of fast fibre mitochondria and IMCL are also similar in GT1 and GT2. (E) Similarly, post-training increases in amounts of mitochondria as indicated by the activities of CS and OXPHOS enzymes do not significantly diverge between GT1 and GT2. GT1 (n = 13), GT2 (n = 15); data given as means ± SE; * intergroup differences significant at *p < 0*.*05*.

Immunolabelling for fibre type-specific MHC isoforms served to analyse the relative proportion of slow (type I), fast (type II) and slow-fast hybrid muscle fibres within the biopsies (Fig 1A and 1B). On average, 773 muscle fibres per sample were analysed (range 308–1944). At baseline, the mean proportion of slow fibres in GT1 was 50.5 ± 2.5% and in GT2 59.9 ± 2.8% (*p = 0*.*02*, ANOVA, Table 2). After the training programme, the mean percentage of slow fibres in GT1 increased by 8.9 ± 2.6% which corresponds to a significant increase of 19% of the initial values (*p < 0*.*01*, Table 2, Fig 2A). By contrast, the relative numbers of slow fibres in GT2 decreased by 1.5 ± 2.6% (*ns*, Table 2, Fig 2A). Thus, only the control subjects that carry the major allele were capable of increasing the slow fibre frequency due to the training stimulus (*p = 0*.*01*, ANOVA, Table 2). The proportion of slow-fast hybrid fibres was very low (about 1.5% in both groups, Table 1) and showed no significant change after training (Table 2).

### Capillarization

Quantification of capillaries based on anti-CD31 immunostaining (Fig 1C) revealed that at baseline, there are no significant differences in the mean number of capillaries per slow fibre in GT1 and GT 2 (6.37 ± 0.15 and 6.61 ± 0.20, Table 1). In both groups, the 10 weeks lasting endurance training induced a significant increase in capillary density. Capillary density increased by 1.19 ± 0.10 (+19.0% of the baseline values, p < 0.001, Fig 2B) in GT1, and by 1.17 ± 0.10 (+18.4%, p < 0.001, Fig 2B) in GT2 (Table 2). The training evoked changes in capillary supply are similar for both groups (*p = 0*.*60*).

### Mitochondria and intramyocellular lipid content (IMCL)

At baseline, there were no significant differences between GT1 and GT2 regarding the fine structural variables. Slow fibres of GT1 and GT2 contained on average 6.97 ± 0.36% and 7.90 ± 0.33% mitochondria and 1.32 ± 0.21% and 1.07 ± 0.16% IMCL, respectively (Table 1). Similarly, the relative proportions of these intracellular components were not significantly different in fast fibres: mean volume densities of mitochondria and IMCL were at 3.35 ± 0.19% and 0.41 ± 0.10%, respectively, in GT1, and at 3.67 ± 0.25% and 0.37 ± 0.05%, respectively, in GT2 (Table 1).

After the training intervention, the slow fibres of GT1 exhibited an increase by 2.99 ± 0.50% in mitochondrial content (+46% of initial values, *p = 0*.*002*, Fig 2C), and an increase by 1.14 ± 0.26% in IMCL (+139% of baseline values, *p < 0*.*001*, Fig 2C). Rather similar increases in these two variables were detected in slow fibres of GT2. Mitochondria increased by 2.46 ± 0.66% (+33%, *p < 0*.*001*, Fig 2C), and IMCL by 0.68 ± 0.28% (+88%, *p = 0*.*03*, Fig 2C).

The results show clearly that the training programme conducted in this study promotes a change towards slow fibres with higher aerobic capacity. This change does apparently not depend upon genotype (Table 2).

We also aimed to detect whether there are genotype-dependent effects on the volume densities of mitochondria and IMCL in fast fibres. In this respect, the data show clearly that this is also not the case. In fast fibres of both GT1 and GT2, the endurance training led to a strong increase in mitochondrial content by 2.35 ± 0.47% (+64% of initial values, *p < 0*.*001*, Fig 2D) and by 1.24 ± 0.41% (+44%, *p = 0*.*009*, Fig 2D), respectively (Table 2). This was accompanied by increases in content of IMCL by 1.14 ± 0.26% (+234%, *p = 0*.*23*, Fig 2D) and by 0.68 ± 0.28% (+400%, *p = 0*.*83*, Fig 2D) in GT1 and GT2, respectively (Table 2).

### Mitochondrial enzyme activities

The 10 wks cycling training caused a significant increase in citrate synthase activity (+ 108 ± 28 Units / g protein; *p < 0*.*001*, Table 2, Fig 2E), which is used as a marker for the mitochondrial mass. This is in agreement with the significantly higher proportions of slow and fast fibre mitochondria found in the stereological analysis (Table 2). Complex II was significantly higher in GT1, while only a trend towards higher values was observed for GT2 (Table 2). A significant elevation of total COX (= complex IV), as well as of the activity of the citrate synthase-normalized COX is obvious in both groups. Normalized complex I values are lower in GT1. In both groups, no significant changes were observed concerning the mtDNA copy number.

MtDNA copy number, total enzymatic activities as well as to the citrate synthase normalized values from biopsy tissue samples taken prior to training did not differ significantly between the groups (Table 1). The increase in total enzymatic activities of the citrate synthase and the OXPHOS complexes was more pronounced in GT1 compared to GT2 (Fig 2E, Table 2).

## Discussion

The present results provide a comprehensive characterization of exercise-induced changes in muscle structure and mitochondrial function in humans carrying the Gly482Ser SNP in the PGC-1α gene. The findings supplement our previous study in which we have shown that this SNP decreases the effectiveness of aerobic exercise training at submaximal performance levels [38]. Here, we present quantitative data from analysis of biopsies from *vastus lateralis* muscle that demonstrate that the carriers of the Gly482Ser SNP (study group GT2), in contrast to the control group GT1, lacked a training-induced increase in content of slow contracting oxidative fibres (Fig 2A, Table 2). By contrast, all other investigated variables (capillary supply, densities of mitochondria and IMCL, and mitochondrial enzyme activities) did not differ between carriers and controls and increased similarly in both groups.

The observations made on the general effects of endurance training on the human skeletal muscle are in agreement with a variety of previous studies. Regarding the promotion of fast-to-slow fibre type conversion, it is long established that high intensity endurance training, whether on the bicycle ergometer or by long distance running is able to induce increases in slow/type I fibre content in the range from 12 to 17% [58,59]. Subjects that underwent high intensity endurance training for a decade or more were shown to have nearly twice as many slow/type I fibres in the *vastus lateralis* muscle than non-trained subjects [60]. However, it should be noted that other work reported no change of slow fibre proportions in response to endurance training [61,62]. Such heterogeneous results may be explained by a variety of factors, including differences in the intensity and duration of the stimulus, age and lifestyle history of the participants, and even methodology of fibre type analysis. Thus, the present data suggest that the exercise stimulus applied was long and intense enough to induce a clear shift from fast/type II to slow/type I in the untrained G1 control subjects which are not affected by the Gly482Ser SNP in the PGC-1α gene. Our data on mitochondrial volume density and OXPHOS activity (Fig 2C–2E, Table 2) are also in good agreement with previous reports that endurance exercise induces increases in mitochondrial numbers [63,64] and elevated levels of mitochondrial respiratory complex enzyme activities [63,59,61,65,23].

A similar background exists for the findings that endurance exercise provokes an increase in IMCL content in both slow/type I and fast/type II fibres, and an increment in capillary density (Fig 2B–2D, Table 2). Higher amounts of IMCL have been established in the muscles of endurance-trained athletes [66,67], and in those of overweight to obese, insulin-resistant, older subjects. After moderate exercise training, the latter could improve their IMCL content by 21% [68]. The lipid droplets are usually aggregated in the vicinity of mitochondria and are regarded as energy source during prolonged exercise [13]. It is also well known that human skeletal muscle responds to endurance training by the formation of new capillaries [69,64,61].

A valuable extension in the present understanding of the implications of SNP Gly482Ser emerges in relation to the observation that the carriers of this SNP fail to respond to endurance training by a fast-to-slow conversion in the fibre type proportions while all other training-sensitive variables are equally responsive in carriers and controls. This demonstrates that the role of Gly482Ser in training response is highly selective, thus allowing to suggest that it is intimately connected with, and confined to, the regulatory effectiveness of myocyte enhancer factor 2 (MEF2). This transcription factor is a key regulator of slow muscle identity [70]. Previous work has shown that the Gly482Ser SNP changes the amino acid sequence in the MEF2 binding site of the PGC-1α protein and causes an impaired interaction with MEF2C [32]. MEF2 proteins are activated through the calcium-regulated calcineurin signaling pathway [70,71]. When overexpressed, MEF2C promotes the formation of slow fibres, thus enhancing running endurance in mice [72]. Genetic deletion of *Mef2c* has been shown to block activity-dependent (exercise-induced) fast-to-slow fibre type transition [72]. This is in line with the proposed role of PGC-1α in such transitions. Muscle-specific overexpression of PGC-1α has been shown to evoke a transition of glycolytic type II in oxidative type I fibres [18]. This shift is initiated by the formation of a PGC-1α/MEF2 transcription complex which then activates the expression of slow muscle genes [73]. Evidence in mice suggests that a similar change to slow type I fibres is mediated by PGC-1α together with the orphan nuclear receptor estrogen-related receptor-α (ERR-α) via up-regulation of HIF2α [74]. Handschin et al. [19] have shown that PGC-1α deficient mice display a significant shift from slow oxidative type I and fast oxidative IIA toward fast glycolytic type IIX and IIB fibres, resulting in a reduced endurance capacity. The results of the present work provide strong support for the view that impairment of the MEF2 binding site in PGC-1α evoked by the Gly482Ser amino acid change is the key factor behind the absence of fast-to-slow fibre type conversion in the endurance training response of the carriers of this SNP.

From the present results it is likely that it is this deficiency in exercise-induced fast-to-slow fibre type transformation which explains the reduced aerobic performance level of persons with the Gly482Ser SNP [35,33,30,36–38]. The impaired MEF2 binding to PGC-1α may then also explain the observation that carriers of the Gly482Ser SNP exhibit a reduced insulin sensitivity [33,34] and a higher risk for metabolic syndrome and type 2 diabetes [35,33,30,36,37]. This because MEF2 is required for the muscle-specific expression of the insulin-responsive glucose transporter 4 (GLUT4) [75,76]. This protein is located in the muscle cell membrane and mediates the insulin-stimulated glucose transport into the muscle cells thereby reducing blood glucose levels [77].

In contrast to this, our results demonstrate that there is no adverse influence of SNP Gly482Ser on the training response of mitochondrial density, CS and OXPHOS complex activity, and mtDNA copy number, content of intracellular lipid (IMCL) and capillary density. This despite the fact that PGC-1α is known to be a key regulator of mitochondrial biogenesis [18,16,78], and is also involved in the formation of lipid droplets [79,80] and capillaries [81,82,78]. However, the essential difference to PGC-1α dependent fibre type conversion is that these processes depend upon interactions of PGC-1α with coactivators which bind to areas of the protein that are all located outside the MEF2 binding site. A domain between amino acids 200 and 400 interacts with the nuclear receptors PPARγ and NRF-1 [83], which are considered as master regulators of mitochondrial biogenesis [84]. PGC-1 binds to and activates the transcriptional function of NRF-1 on the promoter for TFAM, a direct regulator of mitochondrial DNA replication and transcription [16]. Another domain that predominantly binds to nuclear hormone receptors such as ERR-α, PPARs, RXR, glucocorticoid receptor, HNF4, and probably others, is an LXXLL sequence in the N-terminal region of PGC-1α [83]. This sequence is necessary for the coactivation of the nuclear receptor liver x receptor α (LXRα) [85]. The transcription complex of LXRα and PGC-1α then activates fatty acid synthase (FAS), a multifunctional enzyme that catalyzes all reactions required for the *de novo* biosynthesis of lipid [79]. The binding site of the nuclear receptor ERR-α is also in the LXXLL region of PGC-1α [83]. The transcription complex formed by ERR-α and PGC-1α induces the expression of VEGF, a potent stimulator of angiogenesis [86,81].

An at first sight surprising result is that the carriers of SNP Gly482Ser had, despite their disability to activate fast-to-slow fibre type transition in response to training, at baseline significantly more slow oxidative type I fibres than the controls. Interestingly, a similar surplus of slow type I fibres compared to genetically normal controls is known from analyses of fibre type proportions in the *gastrocnemius*, *plantaris* and *soleus* muscles of PGC-1α knock-out mice [78,87]. The reason for this is as yet undetermined. However, the very presence of slow type I fibres in such mice at least demonstrates that PGC-1α is not mandatory for embryonic/fetal slow fibre formation and for the subsequent maintenance of this fibre type. Rather, our data suggest that PGC-1α is involved in exercise-induced fibre type transformation.

In conclusion, the present study provides evidence for the first time that the Gly482Ser SNP in *PGC-1α* disables exercise-induced fast-to-slow fibre type transformation while all other important variables, such as capillary supply, mitochondrial density, mitochondrial enzyme activities and intramyocellular lipid content, exhibit clear responses to the training intervention. This may be a key element to explain the overall reduced effectiveness of aerobic exercise training at submaximal performance levels [38]. Beyond confirming the previously established role of PGC-1α in muscle fibre type transformation, the work adds to our understanding of the molecular mechanisms by which endurance exercise training changes the phenotype of human skeletal muscle, and it highlights the importance of analysing disease related SNP alleles in the development of future strategies of prevention and therapy.
